# Nickel’s carcinogenicity: the need of more studies to progress

**DOI:** 10.1186/s40779-024-00509-8

**Published:** 2024-01-25

**Authors:** Consolato M. Sergi

**Affiliations:** 1 Department of Laboratory Medicine and Pathology, Children’s Hospital of Eastern Ontario (CHEO), University of Ottawa, Ottawa, ON K1N 6N5 Canada; 2 Department of Laboratory Medicine and Pathology, Stollery Children’s Hospital, University of Alberta, Edmonton, AB T6G 2B7 Canada

**Keywords:** Nickel, Alloy, Carcinogenicity, International Agency for Research on Cancer (IARC)

Dear Editor,

On March 8–15, 2022, a board of international scientists assembled in Lyon to evaluate the carcinogenicity of cobalt metal, cobalt(II) salts, antimony trioxide, and weapons-grade tungsten alloy harboring nickel and cobalt [1]. The 131st International Agency for Research on Cancer (IARC) Monograph is the result of a 6–9-month work of perusing the literature, slide evaluation, data interpretation, and interim meetings. The assessment of cobalt, antimony, and nickel-containing alloys will have tremendous consequences for the industry, health, and defense departments [1].

Armor-penetrating projectiles utilize tungsten alloys of weapons-grade quality, consisting of 91–93% tungsten, 2–4% cobalt, and 3–5% nickel. Inhalation of hazardous substances can occur because of occupational exposure in weapons production. Both military personnel and civilians may encounter nickel-containing metal aerosols that are generated during the firing or impact of weapons. Long-term exposure to residual embedded fragments from munitions can pose significant hazards. The available exposure data were limited, nevertheless, the evidence supporting the development of cancer in experimental animals was deemed satisfactory.

Rodents developed rhabdomyosarcoma after being implanted with weapons-grade tungsten alloy in their muscles [2, 3]. This tumor is a malignant mesenchymal neoplasm with skeletal muscle differentiation at the implantation site. The Working Group did not have access to any studies on cancer in humans, and the evidence regarding the mechanisms involved was deemed insufficient. Exposure of humans to settings contaminated with nickel, such as those related to nickel electroplating, refining, and welding, is not insignificant and can lead to different pathological effects, including skin allergies, lung fibrosis, and respiratory tract tumors. In the Kalinich et al. [2] study, 4 clusters of F344 rats were embedded with pellets. The experimental groups consisted of tantalum, low-dose tungsten alloy, high-dose tungsten alloy, and nickel. At about 16–20 weeks post-implantation, neoplasms were detected at the pellet grafting site in the tungsten alloy and nickel-implanted rats. Rhabdomyosarcomas were observed in rats exposed to high doses of tungsten alloy. In another study, B6C3F1 mice have been utilized [3]. The implanted animals formed rhabdomyosarcomas. The occurrence of this neoplasm at the site of implantation showed a substantial rise in the 6-, 12-, and 24-month post-implantation groups, when compared with tantalum-implanted group and sham control group. WNiFe as well as sham and tantalum-implanted mice did not develop neoplasms, while all nickel-implanted mice showed rhabdomyosarcomas as early as 3 months post-implantation.

Nickel and nickel compounds can be encountered through inhalation, ingestion, and skin contact in facilities involved in the production of metallic nickel, nickel compounds, and nickel alloys, as well as in downstream activities including battery manufacturing and electroplating processes [e.g., electric automobiles or vehicles (EVs)]. In the world, the global shift towards sustainable energy is pretty evident. There is a significant increase in the manufacturing of batteries specifically designed for electricity storage, particularly for EVs. The demand for nickel, namely for usage in EVs, is expected to increase exponentially. Due to the energy-intensive nature of extracting metals and minerals for batteries, battery production has a distinct environmental impact. Mining nickel, a crucial element for EVs, can lead to significant air pollution, disastrous water contamination, and habitat devastation. Additionally, individuals might be exposed to nickel through direct contact with body-piercing materials, buttons, and jewelry and the trend for such objects is increasing worldwide.

Moreover, the precise processes underlying nickel-induced carcinogenesis have not yet been fully elucidated and require further studies. The most effective method of delivering nickel(II) dosages into the cells is by the process of phagocytosis, where cells engulf and ingest nickel-containing dust particles that have low solubility. The genotoxicity of nickel(II) can be worsened by the production of reactive oxygen species that damage DNA, as well as by this metal’s ability to hinder DNA repair. The wide range of epigenetic alterations caused by nickel(II) involves changes in gene expression caused by DNA hypermethylation and histone hypoacetylation. Moreover, it appears that the activation or inhibition of transcription factors is implicated. Understanding the biochemical basis of nickel(II) interactions with intracellular targets/ligands, organic compounds, and oxidants substantially enhances the research on the harmful consequences of nickel. Research is being conducted to identify potential nickel(II) targets that are relevant to the development of cancer and to forecast the harmful consequences produced by exposure to nickel [4–6]. In conclusion, the research on nickel carcinogenesis should focus on the development of therapies that can impede or prevent the interactions between nickel(II) and essential molecules [7].

F. William Sunderman, MD, Ph.D., one of the fathers of Laboratory Medicine, devoted his scientific life to the toxicology and carcinogenicity of nickel [7, 8]. Sunderman’s advocacy of transparent and reproducible investigation is a prolegomenon to the current IARC evaluation. We pledge that new studies are on the horizon to strengthen and coruscate the nickel carcinogenicity or refuse it.

## Data Availability

All data are available online and at the IARC/WHO repositories.

## Abbreviations

IARC: International Agency for Research on Cancer
EVs: Electric automobiles or vehicles
